# Association between Free Light Chain Levels, and Disease Progression and Mortality in Chronic Kidney Disease

**DOI:** 10.3390/toxins5112058

**Published:** 2013-11-08

**Authors:** Lucie Desjardins, Sophie Liabeuf, Aurélie Lenglet, Horst-Dieter Lemke, Raymond Vanholder, Gabriel Choukroun, Ziad A. Massy, European Uremic Toxin (EUTox) Work Group

**Affiliations:** 1 INSERM U1088, UFR de Médecine et Pharmacie, Université de Picardie Jules Verne, Amiens 80054, France; E-Mails: desjardins.lucie@chu-amiens.fr (L.D.); liabeuf.sophie@chu-amiens.fr (S.L.); terrier.lenglet.aurelie@chu-amiens.fr (A.L.); choukroun.gabriel@chu-amiens.fr (G.C.); 2 Clinical Research Centre—Division of Clinical Pharmacology, Amiens University Hospital and the Jules Verne University of Picardy, Amiens 80054, France; 3 Division of Pharmacy, Amiens University Hospital, Amiens 80054, France; 4 EXcorLab GmbH, Obemburg 63785, Germany; E-Mail: horstdieter.lemke@excorlab.de; 5 Nephrology Section, Department of Internal Medicine, University Hospital, Ghent 9000, Belgium; E-Mail: raymond.vanholder@ugent.be (R.V.); 6 Division of Nephrology, Amiens University Hospital, Amiens 80054, France; 7 Division of Nephrology, Ambroise Paré Hospital, University of Versailles-Saint-Quentin-en-Yvelines, Boulogne-Billancourt 92100, France

**Keywords:** uremic toxins, free light chain, chronic kidney disease

## Abstract

Immunoglobulin free light chains (FLCs) form part of the middle molecule group of uremic toxins. Accumulation of FLCs has been observed in patients with chronic kidney disease (CKD). The aim of the present study was to measure FLC levels in patients at different CKD stages and to assess putative associations between FLC levels on one hand and biochemical/clinical parameters and mortality on the other. One hundred and forty patients at CKD stages 2-5D were included in the present study. Routine clinical biochemistry assays and assays for FLC kappa (κ) and lambda (λ) and other uremic toxins were performed. Vascular calcification was evaluated using radiological techniques. The enrolled patients were prospectively monitored for mortality. Free light chain κ and λ levels were found to be elevated in CKD patients (especially in those on hemodialysis). Furthermore, FLC κ and λ levels were positively correlated with inflammation, aortic calcification and the levels of various uremic toxins levels. A multivariate linear regression analysis indicated that FLC κ and λ levels were independently associated with CKD stages and β2 microglobulin levels. Elevated FLC κ and λ levels appeared to be associated with mortality. However, this association disappeared after adjustment for a propensity score including age, CKD stage and aortic calcification. In conclusion, our results indicate that FLC κ and λ levels are elevated in CKD patients and are associated with inflammation, vascular calcification and levels of other uremic toxins. The observed link between elevated FLC levels and mortality appears to depend on other well-known factors.

## 1. Introduction

Uremic toxins are retention solutes that accumulate in the blood of patients with kidney failure. These molecules contribute to a variety of metabolic and functional disorders (e.g., impaired immune responses). Immunoglobulin light chains have a mean molecular weight of 25,000 Daltons for monomers and approximately 50,000 Daltons for dimers and are considered to be members of the middle molecule family of uremic toxins [1].

Light chains are synthesized by plasma cells; two light chains pair with two heavy chains to form the various classes of immunoglobulins [2]. The concentration of free light chains (FLCs) can be used to monitor the activity of the adaptive immune system [3]. Plasma cells normally produce slightly more light chains than heavy chains, and the excess light chains are then either excreted or catabolized by the kidney [4]. It is thought that light chains (and particularly FLCs) have a role in kidney disease. In chronic kidney disease (CKD), light chains are filtered by the glomeruli and then reabsorbed by the proximal tubuli [5]. Indeed, it is known that the renal disease associated with monoclonal gammopathy involves the deposition of monoclonal immunoglobulin deposits in the kidney’s extracellular matrix [6]. Moreover, direct injury of the renal tubular epithelium by the monoclonal protein is mostly seen in multiple myeloma patients who develop myeloma cast nephropathy (also known as Bence Jones cast nephropathy), with the formation of giant cells around casts present in distal tubules [7].

In CKD patients, low renal clearance of polyclonal FLC induces an elevation of FLC kappa (κ) and lambda (λ) levels. Indeed, two previous studies have shown that elevated serum concentrations of FLCs are correlated with parameters of kidney function like creatinine and cystatin C [5,8]. Hutchison *et al*. showed that FLC κ and λ concentrations rise as kidney function declines and are highest in patients on hemodialysis. In the latter population, the urine FLC concentrations varied according to the type of renal disease, the CKD stage and the albuminuria value [5].

However, data related to the potential toxicity (in terms of cardiovascular disease and mortality) of FLC accumulation in CKD patients are scarce. In one study, Haynes *et al*. failed to observe a significant association between a monoclonal excess of FLCs and risk of mortality and end-stage renal disease (ESRD) after adjustment for baseline estimated glomerular filtration rate (eGFR) in 364 CKD patients [8]. 

Therefore, the objectives of the present study were to (i) evaluate FLC κ and λ levels in patients at different CKD stages and (ii) assess the link between FLC κ and λ levels and biochemical and clinical parameters (including vascular calcification) and (iii) probe the putative association between FLC κ and λ levels and mortality.

## 2. Results

**Figure 1:**
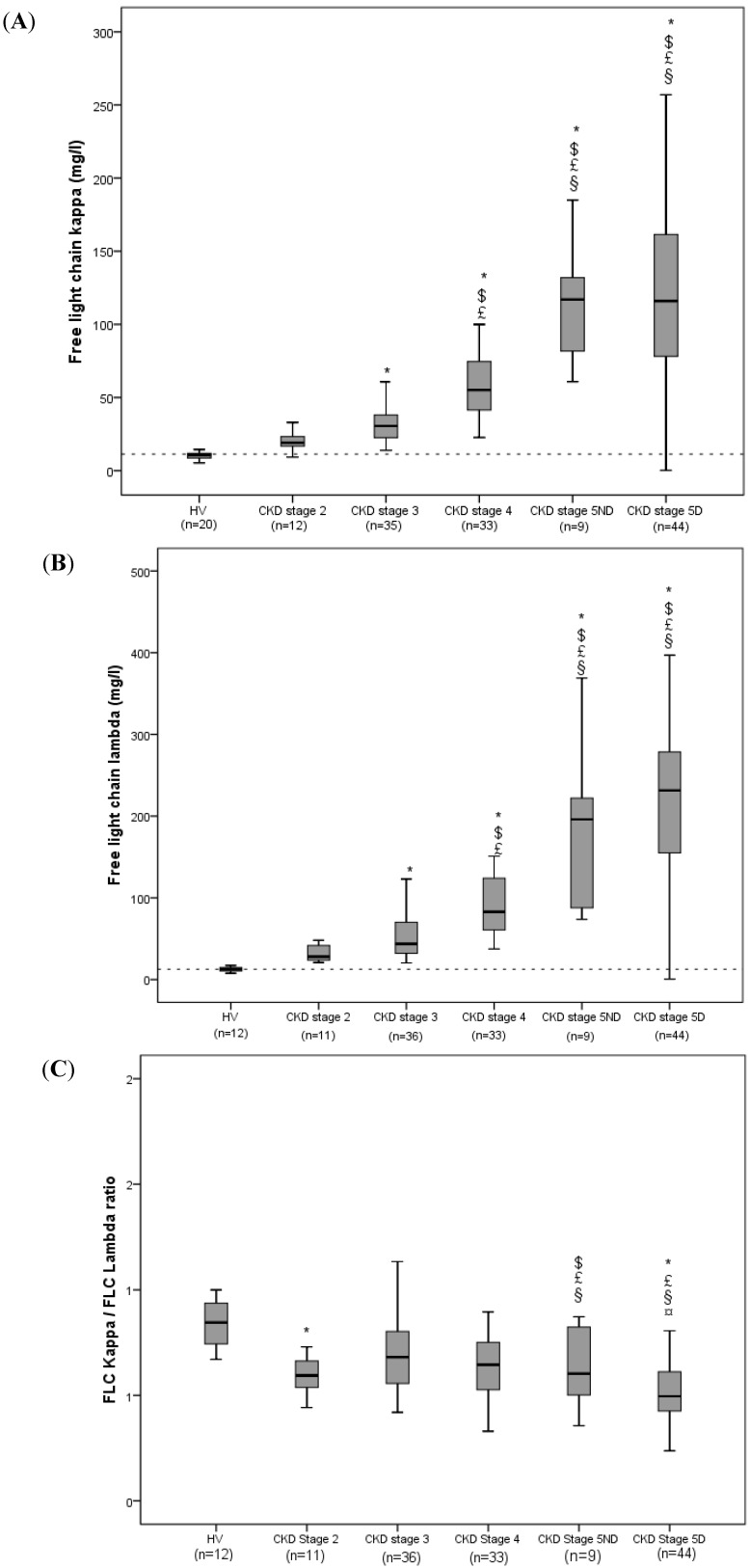
Levels of free light chain κ (**A**), λ (**B**) and κ/λ (**C**) as a function of the chronic kidney disease (CKD) stage. * *p* < 0.05 *vs*. healthy volunteers (HVs); $ *p* < 0.05 *vs*. CKD stage 2; £ < 0.05 *vs*. CKD stage 3; § *p* < 0.05 *vs*. CKD stage 4; ¤ *p* < 0.05 *vs*. CKD stage 5. CKD: chronic kidney disease. The dotted lines indicate the reference value derived from HVs (11.3 mg/L for FLC κ and 12.6 mg/L for FLC λ).

The distribution of FLC κ and λ levels by CKD stage is shown in Figure 1A–C. Mean FLC κ and λ levels were significantly higher in the total study population (74.4 ± 59.4 mg/L and 48.3 ± 20.3 mg/L for FLC κ and λ levels, respectively) than in healthy volunteers (11.3 ± 4.7 mg/L and 12.6 ± 3 mg/L, respectively) (*p* < 0.001). Furthermore, FLC κ and λ levels rose progressively with the CKD stage. The initiation of hemodialysis does not affect FLC κ and λ levels. When the analysis was restricted to the 96 predialysis patients enrolled in the study, significant inverse exponential relationships between FLC κ and λ levels (*r^2^* = 0.474 and *r^2^* = 0.433, respectively) and eGFR were found.

Table 1 and Table 2 summarize the study population’s main demographic, clinical and biochemical characteristics as a function of the median FLC κ and λ levels.

Patients with a FLC κ level greater than or equal to the median value (55.2 mg/L) presented a higher aortic calcification score (the X-ray-derived score for FLC κ and λ and the CT-derived score for FLC κ) and higher phosphate, triglyceride, iPTH, urea and IL-6 levels than patients below the median value did. When the population was divided according to the median λ FLC level (86.1 mg/L), bivariate comparisons yielded essentially the same results as described above for FLC κ.

Moreover, patients with higher FLC κ and λ levels also had higher levels of protein-bound uremic toxins (free IS and PCS) and β2M (representative of “middle molecules”).

**Table 1:** Clinical and demographic characteristics of the study population.

	Total (*n* = 133)	FLC κ < 55.2 mg/L (*n* = 67)	FLC κ ≥ 55.2 mg/L (*n* = 66)	*p*	FLC λ < 86.1 mg/L (*n* = 67)	FLC λ ≥ 86.1 mg/L (*n* = 66)	*p*
Age, years	67 ± 12	67 ± 12	68 ± 13	0.687	68 ± 12	67 ± 13	0.619
Male gender, *n* (%)	82 (61.7)	42 (62.7)	40 (60.6)	0.859	43 (64.2)	39 (59.1)	0.595
Body mass index, kg/m^2^	28.3 ± 6.2	28.9 ± 6.8	27.2 ± 5.3	0.102	28.7 ± 6.5	27.5 ± 5.7	0.284
History of CVD, *n* (%)	43 (32.3)	20 (29.9)	23 (34.8)	0.581	21 (31.3)	22 (33.3)	0.854
Systolic blood pressure, mmHg	154 ± 27	149 ± 23	158 ± 30	0.057	152 ± 26	155 ± 28	0.580
Diastolic blood pressure, mmHg	81 ± 12	82 ± 11	80 ± 14	0.374	82 ± 12	80 ± 12	0.246
Pulse wave velocity, m/s	14.6 ± 3.85	14.2 ± 3.6	15.2 ± 4.1	0.152	14.6 ± 3.7	14.8 ± 4.1	0.779
CKD stage, *n* (%)				<0.001			<0.001
2	12 (9)	12 (17.9)	0 (0)		10 (14.9)	1 (1.5)	
3	35 (26.3)	31 (46.3)	4 (6.1)		32 (47.8)	4 (6.1)	
4	33 (24.8)	17 (25.4)	16 (24.2)		19 (28.4)	14 (21.2)	
5ND	9 (6.8)	0 (0)	9 (13.6)		2 (3)	7 (10.8)	
5D	44 (33.1)	7 (10.4)	37 (56.1)		4 (6)	40 (60.6)	
CT aortic calcification score, %	3.02 ± 3.02	2.31 ± 2.59	3.74 ± 3.27	0.008	2.52 ± 2.72	3.55 ± 3.35	0.065
Coronary calcification score, AUs	604.2 ± 1230.4	400.4 ± 553.2	838.3 ± 1762.2	0.143	451.2 ± 710.3	737.8 ± 1650.5	0.283
X-ray aortic calcification score	6.25 ± 6.55	4.43 ± 5.6	8.16 ± 7.01	0.002	4.33 ± 4.66	8.45 ± 7.58	<0.001

Abbreviations: FLC, free light chain; CVD, cardiovascular disease; CT, computed tomography; ND, not on dialysis; D, on dialysis; AU, Agatston units; HUs, Hounsfield units.

**Table 2:** Biochemical characteristics of the study population.

	Total (*n* = 133)	FLC κ < 55.2 mg/L (*n* = 67)	FLC κ ≥ 55.2 mg/L (*n* = 66)	*p*	FLC λ < 86.1 mg/L (*n* = 67)	FLC λ ≥ 86.1 mg/L (*n* = 66)	*p*
Total calcium, mmol/L	2.29 ± 0.19	2.32 ± 0.15	2.26 ± 0.21	0.065	2.32 ± 0.14	2.27 ± 0.22	0.079
Phosphate, mmol/L	1.29 ± 0.45	1.13 ± 0.37	1.42 ± 0.48	<0.001	1.12 ± 0.27	1.42 ± 0.53	<0.001
Triglycerides, mmol/L	2.08 ± 1.38	1.83 ± 1.01	2.33 ± 1.57	0.035	1.71 ± 0.85	2.34 ± 1.63	0.060
Cholesterol, mmol/L	4.89 ± 1.18	4.9 ± 1.11	4.81 ± 1.23	0.595	4.97 ± 0.99	4.77 ± 1.34	0.322
HDLc, mmol/L	1.34 ± 0.48	1.39 ± 0.47	1.2 ± 0.5	0.232	1.41 ± 0.45	1.28 ± 0.98	0.131
LDLc, mmol/L	2.63 ± 0.9	2.7 ± 0.91	2.54 ± 0.92	0.311	2.77 ± 0.77	2.45 ± 0.98	0.044
iPTH, pg/mL	136.8 ± 137.2	90.4 ± 79.8	185.7 ± 168.7	<0.001	86.6 ± 70.7	187.8 ± 169.9	<0.001
Urea, mmol/L	20.43 ± 10.56	15.58 ± 8.31	24.82 ± 10.63	<0.001	15.98 ± 8.51	24.75 ± 10.76	<0.001
25 (OH) vitamin D, ng/mL	20.4 ±13.6	20.3 ± 12.1	20.9 ± 15.2	0.785	20.9 ±12.4	20.5 ± 14.9	0.862
1,25 (OH)_2_ vitamin D, pg/mL	11.4 ± 10.7	13.6 ± 11	9.3 ± 10.3	0.054	14.5 ± 11.9	7.3 ± 6.8	<0.001
eGFR, mL/min, 1.73 m^2^	35.1 ± 18.9	43.1 ± 18.3	20.3 ± 8.5	<0.001	41.3 ± 18.3	21.2 ± 11.7	<0.001
IL6, pg/mL	5.26 ± 7.89	3.57 ± 4.9	6.9 ± 9.97	0.025	3.19 ± 3.55	7.42 ± 10.4	0.004
CRP, mg/L	11.2 ± 23.89	8.34 ± 23.39	14.1 ± 25.32	0.175	6.7 ± 10.5	15.7 ± 32.3	0.034
β2 microglobulin, mg/L	13.54 ± 12.51	6.3 ± 7.5	21.3 ± 12.7	<0.001	6.08 ± 6.78	21.1 ± 12.81	<0.001
Free indoxyl sulfate, mg/100 mL	0.08 ± 0.098	0.05 ± 0.06	0.12 ± 0.12	<0.001	0.04 ± 0.06	0.13 ± 0.12	<0.001
Free p-cresyl sulfate, mg/100 mL	0.26 ± 0.51	0.008 ± 0.15	0.45 ± 0.64	<0.001	0.066 ± 0.143	0.482 ± 0.669	<0.001
FLC κ, mg/L	74.36 ± 59.54	31.51 ± 12.79	117.86 ± 56.74	-	-	-	-
FLC λ, mg/L	131.94 ± 117.09	-	-	-	48.34 ± 20.35	216.81 ± 113.6	-

Abbreviations: FLC, free light chain; HDLc, high density lipoprotein cholesterol; LDLc, low density lipoprotein cholesterol; iPTH, intact parathyroid hormone; CRP, *C*-reactive protein.

Univariate correlations with FLC κ and λ levels are presented in Table 3. The FLC κ and λ levels were positively correlated with phosphate, IL6, CRP, triglyceride, PTH, urea, β2M, IS and PCS levels and the X-ray and CT aortic calcification scores. A negative correlation was found for high density lipoprotein (HDL) cholesterol, calcium and 1.25 (OH)_2_ vitamin D levels and eGFR.

**Table 3:** Correlation between free light chain kappa and lambda levels and selected clinical and biochemical characteristics (*n* = 133).

	FLC κ		FLC λ	
	*r*	*p*	*r*	*p*
Age	0.022	0.804	−0.030	0.734
Gender	0.082	0.347	0.016	0.859
BMI	−0.041	0.640	−0.068	0.438
History of CVD	0.076	0.385	0.068	0.438
Systolic blood pressure	0.125	0.152	0.056	0.526
Diastolic blood pressure	−0.097	0.269	-0.148	0.090
Pulse wave velocity	0.093	0.286	0.035	0.687
Calcium	−0.183	0.035	−0.197	0.023
Phosphate	0.376	<0.001	0.356	<0.001
Triglycerides	0.246	0.005	0.215	0.014
Cholesterol	−0.075	0.400	−0.143	0.107
HDLc	−0.186	0.036	−0.241	0.006
LDLc	−0.080	0.369	−0.158	0.075
PTH	0.365	<0.001	0.370	<0.001
Urea	0.546	<0.001	0.508	<0.001
IL6	0.353	<0.001	0.414	<0.001
CRP	0.219	0.011	0.236	0.006
25 (OH) vitamin D	−0.017	0.849	−0.031	0.723
1,25 (OH)_2_ vitamin D	−0.292	0.004	−0.304	0.003
eGFR *	−0.795	<0.001	−0.764	<0.001
Free indoxyl sulfate	0.649	<0.001	0.653	<0.001
Free p-cresyl sulfate	0.573	<0.001	0.606	<0.001
β2 microglobulin	0.838	<0.001	0.823	<0.001
CT scan aortic calcification score	0.278	0.002	0.205	0.023
Coronary calcification score	0.152	0.159	0.117	0.282
X-ray aortic calcification score	0.319	0.001	0.282	0.002

Abbreviations: BMI, body mass index; CVD, cardiovascular disease; HDLc, high density lipoprotein cholesterol; LDLc, low density lipoprotein cholesterol; iPTH, intact parathyroid hormone; CRP, *C*-reactive protein. * eGFR measured for patients at CKD stages 2–5; patients on dialysis were excluded.

It is noteworthy that FLC κ and λ levels were positively correlated with aortic calcification but not coronary calcification. In order to identify clinical biochemical parameters that might be independently associated with elevated FLC κ and λ levels in our CKD population, we performed several multivariate analyses (Table 4) and found that FLC κ and λ levels were indeed independently associated with CKD stages and β2M.

**Table 4:** Multivariate linear regression: variables independently associated with free light chain kappa and lambda (log-normalized) (*n* = 133).

	FLC κ	
	β (95% CI)	*p*
Model 1 ( *R*^2^ = 0.297)		
Age	0.058 (−0.007–0.016)	0.467
Male gender	0.017 (0.260–0.324)	0.828
CKD stage	0.535 (0.237–0.464)	<0.001
Ln IL6	0.013 (0.141–0.163)	0.884
Model 2 ( *R*^2^ = 0.311)		
Age	0.083 (−0.005–0.17)	0.289
Male gender	0.022 (−0.243–0.325)	0774
CKD stage	0.129 (−0.138–0.307)	0.452
Ln IL6	−0.055 (−0.202–0.106)	0.439
Ln β2 microglobulin	0.486 (0.134–0.846)	0.007
	FLC λ	
Model 3 ( *R*^2^ = 0.356)		
Age	0.037 (−0.009–0.014)	0.622
Male gender	−0.034 (−0.360–0.227)	0.653
CKD stage	0.578 (0.294–0.520)	<0.001
Ln IL6	0.092 (−0.063–0.238)	0.254
Model 4 ( *R*^2^ = 0.388)		
Age	0.056 (−0.007–0.015)	0.454
Male gender	−0.030 (−0.344–0.288)	0.688
CKD stage	0.217 (−0.067–0.373)	0.171
Ln IL6	0.018 (−0.139–0.173)	0.834
Ln β2 microglobulin	0.439 (0.119–0.825)	<0.001

Abbreviations: CKD, chronic kidney disease; IL6, interleukin 6; FLC, free light chain.

**Figure 2:**
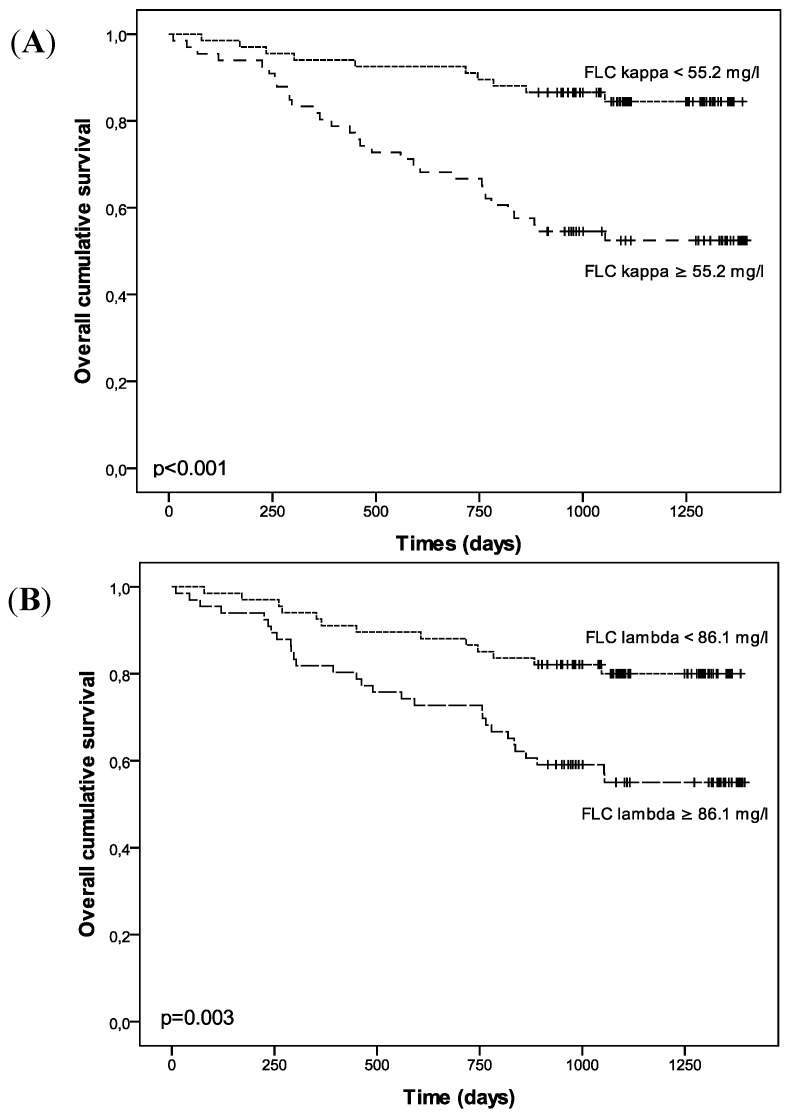
Kaplan–Meyer estimates of overall mortality for patients as a function of the median free light chain κ (**A**) and λ (**B**) levels.

During the follow-up period (mean ± SD duration: 969 ± 374 days; median: 1058; range: 10–1396), there were 42 deaths (including 22 due to cardiovascular events) and seven patients initiated hemodialysis. Elevated FLC κ and λ levels were significantly correlated with overall mortality when treated either as a continuous variable (*p* < 0.001 and *p* = 0.002 respectively, as presented in Table 5 for an unadjusted model) or stratified according to the median level (*p* < 0.001 and *p* = 0.003 respectively; Figure 2). Table 5 details the results of the Cox regression analyses for overall mortality: FLC κ and λ levels were positively associated with overall mortality in unadjusted models but not after adjustment for the CKD stages and a propensity score. When the analysis was restricted to predialysis patients (Table 6), levels of FLC κ (but not FLC λ) were positively associated with overall mortality in an unadjusted modem and in adjusted model including age but not after adjustment for eGFR.

**Table 5:** Multivariate Cox regression analysis of risk factors at baseline for all-cause mortality—Free light chain κ and λ levels entered as the median.

	FLC κ			FLC λ	
Events: *n* = 42	RR (95% CI)	*p*	Events: *n* = 42	RR (95% CI)	*p*
Model 1			Model 1		
Age	1.045 (1.016–1.075)	0.002	Age	1.049 (1.019–1.079)	0.001
FLC kappa	3.836 (1.876–7.845)	<0.001	FLC lambda	2.853 (1.480–5.500)	0.002
Model 2			Model 2		
Age	1.051 (1.020–1.082)	0.001	Age	1.051 (1.022–1.082)	0.001
FLC kappa	1.816 (0.769–4.287)	0.174	FLC lambda	1.349 (0.580–3.141)	0.487
CKD stage	1.561 (1.139–2.318)	0.006	CKD stage	1.530 (1.116–2.096)	0.008
Model 3			Model 3		
Propensity score	138.9 (3.53–5473.62)	0.008	Propensity score	28.3 (5.309–150.8)	<0.001
FLC kappa	1.704 (0.542–5.359)	0.362	FLC lambda	1.437 (0.648–3.186)	0.272

Abbreviations: RR, relative risk; CI, confidence interval; CKD, chronic kidney disease; FLC, free light chain. Propensity score: age, CKD Stage, CT aortic calcification entered as the median.

**Table 6:** Multivariate Cox regression analysis of risk factors at baseline for all-cause mortality in predialysis patients—Free light chain κ and λ entered as the median.

	FLC κ			FLC λ	
Events: *n* = 18	RR (95% CI)	*p*	Events: *n* = 18	RR (95% CI)	*p*
Model 1			Model 1		
FLC kappa	3.052 (1.202–7.751)	<0.019	FLC lambda	1.354 (0.540–3.397)	0.519
Model 2			Model 2		
Age	1.044 (1.001–1.089)	0.047	Age	1.052 (1.007–1.098)	0.022
FLC kappa	2.707 (1.049–6.990)	0.040	FLC lambda	1.327 (0.527–3.337)	0.548
Model 3			Model 3		
Age	1.040 (0.997–1.085)	0.066	Age	1.045 (1.003–1.088)	0.036
FLC kappa	1.218 (0.376–3.946)	0.743	FLC lambda	0.654 (0.223–1.922)	0.440
eGFR	0.959 (0.917–1.002)	0.063	eGFR	0.961 (0.926–0.998)	0.039

Abbreviations: RR, relative risk; CI, confidence interval; FLC, free light chain.

## 3. Discussion

Our present results showed that FLC κ and λ levels were significantly higher in patients at various CKD stages than in healthy controls. The levels increase progressively with CKD stage and are highest in hemodialysis patients. Moreover, FLC κ and λ levels were correlated with other uremic toxins evaluated in this population; there was a strong, independent, positive association with levels of another middle molecule uremic toxin (β2M). Furthermore, FLC levels were positively correlated with aortic but not coronary calcification. In contrast, higher FLC κ and λ levels were significantly associated with mortality in a univariate analysis, but this association was lost after adjustment for renal function and a propensity score including age, CKD stage and the CT calcification score.

Hence, in a cohort of patients at different CKD stages, we confirmed the progressive elevation of FLC κ and λ levels as with increasingly advanced CKD stage. Two previous studies of CKD populations have reported a similar elevation of FLC levels with CKD stage and a strong correlation with markers of renal function [2,9]. Furthermore, Cohen *et al*. demonstrated that currently available haemodialysis or hemodiafiltration treatments are unable to normalize the elevated levels of FLC in ESRD patients [10].

Previous studies of CKD patients did not evaluate the link between FLC and other uremic toxins. In the present study, we evaluated the relationship between FLC κ and λ concentrations and biochemical and clinical parameters and found that FLC κ and λ levels are strongly and positively correlated with levels of other uremic toxins levels, including small molecules (such as phosphate, PTH and urea), middle molecules (such as β2M and IL6) and protein-bound uremic toxins (such as IS and PCS). The strongest correlation was with β2M.

Free light chains have been classified as uremic toxins because there is evidence of direct effects on physiological function in experimental models. Indeed, FLCs are able to modulate the functions of polymorphonuclear leukocytes by inhibiting spontaneous apoptosis [11]. Furthermore, FLCs have been shown to decrease chemotaxis and glucose uptake by neutrophils [12]. Hence, FLCs may have an important role in the immune system in a uremic context. Indeed, our present results show that FLC κ and λ levels are associated with levels of inflammatory markers (IL6 and CRP). Hence, FLCs may have a role in the genesis of the chronic inflammation encountered in CKD patients [13,14].

Lastly, we demonstrated that patients with high levels of FLC κ and λ had an increased risk of mortality. However, this correlation was lost after adjustment for renal function and a calculated propensity score that included age, CKD stage and the CT aortic calcification score. Similarly, Haynes *et al*. did not observe a significant association between excess FLCs and mortality in 364 predialysis patients [8]. Hence, the association between high FLC κ and λ levels and mortality may depend on several other parameters, such as the levels of other uremic toxins (β2M, for example). Indeed, B2M is one of the most extensively studied middle molecule uremic toxins in CKD patients (and especially in dialysis patients, where B2M is the major protein component in dialysis-related amyloidosis). Elevated β2M levels are associated with mortality in hemodialysis [15,16], CKD [17] and kidney transplant patients [18]. Furthermore, a prospective cohort study of 6445 adults aged 20 or more from the Third National Health and Nutrition Examination Survey recently confirmed that B2M levels were better predictors of the mortality risk prediction than conventional measures of kidney function [19].

## 4. Materials and Methods

### 4.1. Ethics Statement

The study was performed in accordance with the principles of the Declaration of Helsinki and in compliance with the International Conference on Harmonization’s guidelines on Good Clinical Practice. The study protocol was approved by the local independent ethics committee (Comité de Protection des Personnes Nord-Ouest II) prior to the initiation of any study-specific procedures. The study was registered with the French health authorities (reference number: 06H3). All patients were provided with full information on the study’s objectives and procedures and gave their written, informed consent to participation.

### 4.2. Patient Selection

Over an 18-month period (from January 2006 to June 2007), a total of 140 Caucasian, prevalent CKD patients were recruited from the Nephrology Department’s outpatient clinic at Amiens University Hospital.

Included patients had to be over the age of 40, with available serum FLC κ and λ results and a confirmed diagnosis of CKD. The latter was defined as being on hemodialysis or having two previous estimated creatinine clearances (calculated according to the Cockcroft and Gault formula <90 mL/min/1.73 m^2^, with an interval of 3 to 6 months). Stage 5D CKD patients had been on chronic hemodialysis three times a week for at least three months. The exclusion criteria consisted of the presence of chronic inflammatory disease, atrial fibrillation, complete heart block, abdominal aorta aneurysm, aortic and/or femoral artery prosthesis, primary hyperparathyroidism, kidney transplantation and any acute cardiovascular event in the three months prior to screening for inclusion. The 140 patients who met all the inclusion criteria and none of the exclusion criteria were included in the present analysis.

### 4.3. Study Protocol

All patients were hospitalized for the day in order to perform laboratory blood tests, blood pressure measurements, a pulse wave velocity (PWV) determination, a lateral lumbar X-ray and a multislice spiral computed tomography (MSCT) scan. For a given patient, all examinations were performed between 9 am and 2 pm on the same day. Hemodialysis patients were seen on a dialysis-free day or, if this was not possible, the morning before the dialysis session. A patient interview focused on comorbidities, the personal disease history and (in particular) any previous vascular events. The patients’ medical files were reviewed in order to identify and record any concomitant medications. For descriptive purposes, patients who reported current or past use of insulin and/or orally administered hypoglycemic drugs were considered to be diabetics. Previous cardiovascular disease was defined as a history of any of the following events: myocardial infarction, stroke, heart failure, angina pectoris, peripheral artery disease and any surgical procedure or percutaneous transluminal angioplasty because of vascular disease.

### 4.4. Laboratory Tests

Blood samples were collected in the morning, before the other investigations were undertaken. Selected assays were performed after the samples had been frozen and stored at −80 °C. Serum calcium, phosphate, albumin, cholesterol, hemoglobin, creatinine (Scr) and C-reactive protein (CRP) levels were assayed in an on-site biochemistry laboratory using standard auto-analyzer techniques (the Modular IIP^®^ system, Roche Diagnostics, Basel, Switzerland). Serum intact parathyroid hormone (iPTH 1-84) was determined in a chemiluminometric immunoassay (Liaison N-tact PTH CLIA^®^, Diasorin, Stillwater, MN, USA). Serum Free FLC κ and λ levels were performed by laser nephelometry (BNProSpec^®^, Siemens Healthcare, Dade Behring, Marburg, Germany).

To determine the concentration of free p-cresylsulphate (PCS), serum samples were deproteinized by heat denaturation and then analyzed by reverse-phase high-performance liquid chromatography (RP-HPLC). The serum concentrations were then determined by fluorescence spectrophotometry (excitation 265 nm; emission 290 nm). The reference value for free PCS in healthy subjects was 0.008 ± 0.009 mg/dL. For the determination of serum indoxyl sulphate (IS) levels, samples were deproteinized by heat denaturation and analyzed with RP-HPLC [20]. The serum concentrations were then determined by fluorescence spectrophotometry (excitation 280 nm, emission 340 nm) using a reference value for IS in healthy controls of 0.113 ± 0.06 mg/100 mL. The plasma concentration of β2 microglobulin (B2M) was measured by immunonephelometry (BNProSpec, Siemens Healthcare Diagnostics GmbH, Eschborn, Germany). 

Serum cystatin C (CysC) levels were also determined by immunonephelometry (BNProSpec analyzer, N latex Cystatin C^®^ assay, Siemens Healthcare Diagnostics GmbH, Eschborn, Germany). In order to assess the true GFR in non-dialyzed patients as accurately as possible, the eGFR combining Scr and CysC measurements was calculated according to the following, recently published “CKD-epi” equation [21]: 177.6 × Scr − 0.65 × CysC − 0.57 × age − 0.20 × (0.82 if female). For descriptive purposes, patients were then classified into CKD stages, according to the National Kidney Foundation’s Kidney Disease Outcomes Quality Initiative guidelines [22].

### 4.5. Pulse Wave Velocity Evaluation

The carotid-femoral PWV was determined automatically with a dedicated device fitted with two pressure probes (Complior Colson, Createch Industrie, Massy, France) and operated by a trained physician, as previously described [23]. Transcutaneously recorded pulse waveforms were obtained simultaneously for the common carotid artery and the femoral artery in the groin. The PWV was calculated as the distance between recording sites measured over the body’s surface (*L*), divided by the time interval (*t*) between the feet of the flow waves (PWV = *L*/*t*); this value was averaged over 10 cardiac cycles [12]. This automated method has been validated previously and has an intra-observer repeatability coefficient of 0.93 and an interobserver reproducibility coefficient of 0.89 [23,24].

### 4.6. Abdominal Aorta Imaging with Plain Radiography

A technique similar to that described by Kauppila *et al*. [25] was used to obtain images of the lower abdominal aorta and thus generate an aortic calcification score. All X-rays were reviewed by two independent investigators and a consensus on the interpretation was reached in all cases. To validate the reproducibility of our vascular calcification measurements, 73 randomly selected radiographies were scored on a blind basis by the two independent investigators.

The very good degree of interobserver agreement on calcification scores was evidenced by a high Pearson correlation coefficient (*r* = 0.925, *p* = 0.01).

### 4.7. Multislice Spiral Computed Tomography

In order to quantify the presence and extent of aortic calcifications, each patient underwent an MSCT scan. All examinations were performed with a 64-detector scanner (Lightspeed VCT^®^, GE Healthcare, Milwaukee, WI, USA).

The volume acquisition started at the aortic hiatus of the diaphragm and ended at the third lumbar vertebra. The scanning parameters were as follows: collimation: 64 × 0.625 mm; slice thickness: 0.625 mm; pitch: 1; gantry rotation speed: 0.5 s/rotation; tube voltage: 120 kV; tube current: 300 mA.

The volume acquisition was analyzed with commercially available software (Volume Viewer^®^ software, GE Healthcare, Milwaukee, WI, USA). The abdominal aorta was segmented manually. In order to reduce errors due to noise, a threshold of 160 UH was applied. The total calcification volume was calculated as the sum of all voxels in the remaining volume. The abdominal aorta calcification score was calculated as follows: ((total calcification volume)/(aorta wall surface area) × 100). The Agatston score was used to quantify coronary calcification [26].

### 4.8. Survival

Death records were established prospectively, by considering all patients included at least twenty months before the study end date (1 January 2010). Each medical chart was reviewed and the cause of death was assigned by a physician based on all the available clinical information. For out-of-hospital deaths, the patient’s general practitioner was interviewed to obtain pertinent information on the cause. Of the 96 predialysis patients, seven patients initiated hemodialysis during the study follow-up period.

### 4.9. Statistical Analyses

Data are expressed as either the mean ± SD, median and range, or frequency, as appropriate. The study patients were stratified according to the median FLC κ concentration (55.2 mg/L) or the median FLC λ concentration (86.1 mg/L). Intergroup comparisons were made using a χ^2^ test for categorical variables and Student’s *t* test or the Kruskal-Wallis test for continuous variables. Spearman correlations were used to identify parameters correlated with FLC levels. Univariate linear regression was performed to evaluate the association between FLC levels and selected demographic, biochemical and clinical variables. Thereafter, a multiple linear regression analysis of the factors selected in the univariate analysis was used to identify those which were independently associated with FLC κ and λ levels. When two variables were strongly correlated, only one variable was retained and separate models were built (Table 4). Similarly, multivariate logistic regressions were performed to identify variables that were independently associated with FLC levels (as categorized by the median). The Kaplan-Meier actuarial curve was used to estimate overall survival relative above and below the median FLC κ and λ level. The log rank test was used to compare survival curves. Univariate analysis and multivariate analyses of mortality were performed by using a Cox proportional hazard model of death as a function of the FLC level. In view of the small size of the present cohort, supplementary Cox regression analyses were performed and included a propensity score adjustment, which considers each individual’s probability of exposure to measure. The propensity score was built on a logistic model that included variables associated with FLC levels (age, CKD stages and CT scan aortic calcification entered as median) as detailed elsewhere [27]. A *p* value ≤ 0.05 was considered to be statistically significant. All statistical analyses were performed using SPSS software (SPSS Inc., Chicago, IL, USA), version 13.0 for Windows (Microsoft Corp., Redmond, WA, USA).

## 5. Conclusions

In conclusion, the present study of patients at various CKD stages confirmed that FLC κ and λ levels are positively correlated with declining kidney function. This is also the first study to show a strong correlation between levels of FLC κ and λ and those of other uremic toxins. In our CKD patients, the observed link between FLC κ and λ levels and mortality appeared to depend on other factors. The use of FLC assays for risk stratification does not appear to be relevant in CKD and so various middle molecule uremic toxins (such as B2M) are indicated.
